# Flora of Kentucky

**Published:** 1833

**Authors:** 


### V. Flora of Kentucky.

Professor Short of Transylvania University, has been for some years engaged in collecting materials for a Flora of his native State. The undertaking is one of such deep interest and great labor, that every Physician of that state should contribute his aid, by collecting and transmitting specimens, together with his observations on the pharmaceutic constitution and medical properties of such of them as belong, or might be introduced into the Materia Medica. Of Professor Short's qualifications for the task he has imposed upon himself, we can speak in terms of unqualified commendation.

